# Histopathological Diagnostic Discrepancies in Soft Tissue Tumours Referred to a Specialist Centre: Reassessment in the Era of Ancillary Molecular Diagnosis

**DOI:** 10.1155/2014/686902

**Published:** 2014-08-05

**Authors:** Khin Thway, Jayson Wang, Taka Mubako, Cyril Fisher

**Affiliations:** Sarcoma Unit, Department of Histopathology, The Royal Marsden NHS Foundation Trust, 203 Fulham Road, London SW3 6JJ, UK

## Abstract

*Introduction*. Soft tissue tumour pathology is a highly specialised area of surgical pathology, but soft tissue neoplasms can occur at virtually all sites and are therefore encountered by a wide population of surgical pathologists. Potential sarcomas require referral to specialist centres for review by pathologists who see a large number of soft tissue lesions and where appropriate ancillary investigations can be performed. We have previously assessed the types of diagnostic discrepancies between referring and final diagnosis for soft tissue lesions referred to our tertiary centre. We now reaudit this 6 years later, assessing changes in discrepancy patterns, particularly in relation to the now widespread use of ancillary molecular diagnostic techniques which were not prevalent in our original study. *Materials and Methods*. We compared the sarcoma unit's histopathology reports with referring reports on 348 specimens from 286 patients with suspected or proven soft tissue tumours in a one-year period. *Results*. Diagnostic agreement was seen in 250 cases (71.8%), with 57 (16.4%) major and 41 (11.8%) minor discrepancies. There were 23 cases of benign/malignant discrepancies (23.5% of all discrepancies). 50 ancillary molecular tests were performed, 33 for aiding diagnosis and 17 mutational analyses for gastrointestinal stromal tumour to guide therapy. Findings from ancillary techniques contributed to 3 major and 4 minor discrepancies. While the results were broadly similar to those of the previous study, there was an increase in frequency of major discrepancies. *Conclusion*. Six years following our previous study and notably now in an era of widespread ancillary molecular diagnosis, the overall discrepancy rate between referral and tertiary centre diagnosis remains similar, but there is an increase in frequency of major discrepancies likely to alter patient management. A possible reason for the increase in major discrepancies is the increasing lack of exposure to soft tissue cases in nonspecialist centres in a time of subspecialisation. The findings support the national guidelines in which all suspected soft tissue tumour pathology specimens should be referred to a specialist sarcoma unit.

## 1. Introduction

Soft tissue tumours are rare with an annual incidence of 2.5 per 100000 population [1] but represent a heterogeneous group of neoplasms that can occur at virtually any anatomic site and thereby occur in the surgical pathology workload of all histopathologists. In the United Kingdom, the National Institute of Clinical Excellence (NICE) and Royal College of Pathologists (RCPath) recommend that patients with a provisional histological and/or radiological diagnosis of bone or soft tissue sarcoma should be referred to specialist multidisciplinary units for evaluation and diagnostic review by a specialist sarcoma pathologist and/or radiologist who are part of a sarcoma multidisciplinary team (MDT) and that there should be a formal system for second opinions and review of difficult cases, with access to diagnostic molecular and cytogenetic facilities [2, 3]. The Royal Marsden Hospital is a tertiary cancer centre whose Sarcoma Unit takes approximately 1500 new histopathology accessions per year, of which about 350 are referral cases. This department has previously published a comparative study of referral and final histological diagnoses of soft tissue tumour specimens referred to the Unit in 2005 [4]. Since then, two developments have occurred: (a) comprehensive adoption of the NICE and RCPath recommendations to routinely refer patients with potential sarcomas to specialist centres and (b) the widespread routine use of ancillary molecular and molecular cytogenetic diagnostic techniques. In this study, we determined areas of diagnostic discrepancy in the reporting of cases of soft tissue tumours referred to a specialist sarcoma unit in 2011, to assess changes in discrepancy patterns in light of these new developments in the interval of 6 years.

## 2. Materials and Methods

### 2.1. Patient Cases

A retrospective reaudit was performed for patients referred with soft tissue lesions to a specialist soft tissue sarcoma unit over a one-year period. The record files within the Department of Histopathology at the Royal Marsden Hospital were examined for a period of 12 months from the 1st of January to the 31st of December 2011. Patients were either surgical or oncological referrals. Referrals to the surgical unit were usually patients with a new histological diagnosis after biopsy, or with recurrent lesions referred for further surgery. Patients were referred to the medical or clinical oncology units for planning of (neo) adjuvant treatment. All second opinion cases (including those sent for pathological opinion, where the patient was not referred to our centre) were excluded, as were cases without referring reports. All cases included in the study had been reviewed by one or both of the specialist soft tissue pathologists (K.T. and C.F.). The material sent comprised either paraffin blocks, unstained slides, and stained slides or a mixture of each. Where blocks or sufficient stained slides were available, additional tests were performed as necessary, including immunohistochemistry, fluorescence in situ hybridisation (FISH), and quantitative real time reverse transcription polymerase chain reaction (RQ-PCR).

### 2.2. Pathology Review

These methods are as previously outlined [4]. Each referring report was compared with the subsequent Sarcoma Unit report for differences in diagnosis and grading. Grading was assigned according to the system by the French Federation of Cancer Centres Sarcoma Group (FNCLCC) [5, 6]. Grading categories were defined as (1) not applicable, (2) not done, (3) no difference in grade, (4) difference by one grade, and (5) difference by two grades. For gastrointestinal stromal tumour (GIST), assessment of potential biological behaviour into low, intermediate, and high risk was also compared as for grading. Tumours, for which the referring pathologist had identified tumour type, performed a mitotic count, and reported on the absence or amount of necrosis but had not given a numerical grade, were retrospectively graded on review and recorded as “graded.” Tumours for which the mitotic count had not been performed, or the presence or absence of necrosis not indicated or where neither had been done, were not retrospectively graded. Tumours which the referring pathologist had assigned as low, intermediate, and high grade were interpreted as grades 1, 2, and 3, respectively. Grading was deemed as “not applicable” (1) in certain sarcomas considered routinely to display aggressive or “high grade” behaviour, (2) in metastatic tumours, (3) in tumours not formally graded, such as dermatofibrosarcoma protuberans (DFSP), (4) in benign lesions, (5) if there was a difference in diagnosis between the referring and specialist unit report, making grading noncomparable, or (6) if there was insufficient material for grading.

### 2.3. Classification of Discrepancies

Major discrepancies were defined as those that could lead to significant change in clinical management, with ensuing under- or overtreatment, and were divided into six groups: (1) malignant > malignant (resulting in significant management change), (2) malignant > benign, (3) benign > malignant, (4) mesenchymal > nonmesenchymal, (5) other (e.g., benign > benign, but resulting in significant management change), and (6) major grading discrepancies, comprising tumours in which there was any interchange of grade between grades 2-3 and grade 1 (as this could lead to management change). Minor discrepancies were divided into those of diagnosis, classification, or grading, but they were those in which the discrepancy was not thought to provoke significant management change. Minor changes in which the discrepancy was purely semantic, or in Sarcoma Unit reports in which subcategorisation was chiefly for special or academic interest (e.g., the addition of a finding of myofibroblastic differentiation within pleomorphic sarcoma), were disregarded. The reasons for discrepancy were analysed, by further assessing reports and reviewing slides where appropriate or possible, to look for sources of error such as interpretation of morphology or immunohistochemistry.

## 3. Results

### 3.1. Patient and Tumour Characteristics

A total of 350 specimens were received from 288 patients in the 1-year period. No review could be made on 2 cases which were excluded from the study: 1 was where only blocks were available and the material was cut out on sectioning and the other was where the wrong slides were sent and no subsequent material was provided. 203 cases were resection specimens and 145 were biopsies (most commonly needle core biopsies). There were 230 cases from district general hospitals, 83 from teaching hospitals, 27 from overseas hospitals, and 8 from private laboratories. There were 167 oncological referrals and 181 surgical referrals. 175 patients were female and 111 were male (ratio 1.57 : 1), and median age at diagnosis was 57.5 years (range 2–96 years). Where available from the gross specimen or cross-sectional imaging, median tumor size was 7 cm (range 1–37 cm). Detailed patient and tumour characteristics are shown in Table 1.

### 3.2. Summary of Discrepancies

Of the 348 cases, 250 (71.8%) were diagnostically concordant or had minimal diagnostic discrepancies. 201 were completely concordant, while 47 were not graded by the referring pathologist, with a grade assigned at our institute. Two cases reported as spindle cell sarcomas were refined at our centre to spindle cell sarcomas with myoid differentiation. Of the 250 cases, 201 were malignant diagnoses, 30 were benign, and 19 were of uncertain or intermediate malignant potential (the majority of which were fibromatosis or inflammatory myofibroblastic tumours (IMT)).

There were 41 minor discrepancies (11.8%) (summarised in Table 2), of which 3 were minor grading differences (i.e., grades 2 to 3 and vice versa). Seven cases were diagnosed as benign lesions, while 31 were malignant diagnoses. There were 57 major discrepancies (16.4%) (summarised in Table 3), of which 10 were reclassified from benign to malignant (including intermediate malignant potential) and 13 were reclassified from malignant (including intermediate) to benign. Overall, these 23 benign-malignant discrepancies accounted for 23.5% of all discrepant cases. Of the remaining 34 cases, 11 were malignant-malignant reclassifications, 1 was a benign-benign reclassification, 8 were major discrepancies in grading, 13 were mesenchymal-nonmesenchymal discrepancies, and 1 was a major discrepancy involving reclassification of a carcinoma. Overall, of the 57 cases, 14 were finally diagnosed as benign, 3 as intermediate malignant potential, and 40 as malignant.

### 3.3. Analysis of Discrepant Cases by Histology

There were 7 total discrepancies involving GIST, with 5 major discrepancies (2 of initial GIST rediagnosed as other spindle cell sarcomas and 1 of initial GIST rediagnosed as fibrous tissue and 2 being major grading discrepancies) and 2 minor grading discrepancies. 19 cases of discrepancy involved leiomyosarcomas, with 5 benign-malignant discordances (3 cases of diagnosis changed from benign entities to leiomyosarcoma, and 2 of leiomyosarcomas changed to benign tumours), 4 major classification discrepancies (involving leiomyosarcomas and other spindle cell tumours), 3 major discrepancies involving grade, and 7 minor classification discrepancies (leiomyosarcomas versus pleomorphic sarcomas with myoid differentiation). Four major discrepancies involved fibromatosis (2 originally diagnosed as benign entities and reclassified as fibromatosis and 2 originally diagnosed as fibromatosis and reclassified as benign). One case was originally diagnosed as IMT but was rediagnosed as Wegener's granulomatosis. Finally, 5 discrepancies involved liposarcomas: 2 major (2 of initial atypical lipomatous tumour/well-differentiated liposarcoma (ALT/WDL) rediagnosed as lipoma, 1 of dedifferentiated liposarcoma (DDL) diagnosed originally as low grade fibromyxoid sarcoma, and 1 of WDL regraded as DDL, grade 2) and 1 minor, originally diagnosed as DDL but reclassified as spindle cell sarcoma after results of FISH.

### 3.4. Contribution of Immunohistochemistry to Diagnoses

Additional immunohistochemistry contributed to change in final diagnosis in 23 cases: in 9/41 minor and 14/57 major discrepancies. Of these 23 cases, the contributory tests had not been performed by the referring centre (rather than these being instances of repeat testing which gave a different pattern or intensity of staining). These were 7 tests for h-caldesmon (for leiomyosarcoma), 3 CDK4 (for WDL/DDL), 2 DOG1 (for GIST), 2 beta-catenin (for fibromatosis), 1 each of CD34 (DFSP), desmin, myogenin (rhabdomyosarcoma (RMS)), p63 (sarcomatoid carcinoma), PSAP (prostatic carcinoma), and TLE1 (synovial sarcoma). In 3 cases, a wide panel of antibodies was used.

### 3.5. Contribution of Molecular and Molecular Cytogenetic Tests to Diagnoses

Of the 348 cases, 50 had FISH or molecular studies performed here. 17 were mutational analyses for* c-kit* and* PDGFRA* genes in GIST, which were performed to guide targeted therapy decisions and which were not contributory to the final histological diagnosis. In 6/50 cases, FISH or RQ-PCR was unsuccessful (technical fails), likely due to DNA and RNA degradation due to differences in tissue fixation in the referral laboratories; 4 of these had no discrepancies between referral and final diagnoses. Two had minor discrepancies: 1 was minor involving grading of GIST, and, in the other, the requested test was for alveolar rhabdomyosarcoma (ARMS). The diagnosis was changed from myoid sarcoma to pleomorphic RMS, and the histologic features were not wholly typical for ARMS, so the failure of the test did not have significant bearing on diagnosis. In the remaining 27 cases, FISH and/or RT-PCR were performed as follows: (1) 12 for* EWSR1* gene rearrangement, of which 9 did not result in discrepancies and 3 resulted in minor discrepancies (1: extraskeletal myxoid chondrosarcoma to poorly differentiated neoplasm, 1: malignant peripheral nerve sheath tumour (MPNST) to clear cell sarcoma, and 1: malignant neoplasm to clear cell sarcoma); (2) 3 for* MDM2* gene amplification, resulting in 2 major discrepancies (1 myxoid liposarcoma to DDL and 1 ALT to lipoma) and 1 minor (DDL to spindle cell sarcoma); (3) 3 for* ALK1 *gene rearrangements with no discrepancies involved; (4) 3 for* SS18-SSX1/2* fusion genes with 2 resulting in no discrepancies and 1 resulting in minor discrepancy to which the test did not contribute (leiomyosarcoma to spindle cell sarcoma); (5) 2 for* FUS-CREB3L1/2* fusion genes with no discrepancies; (6) 2 for* PAX3/7-FOXO1* gene fusions, with 1 resulting in no discrepancies and 1 case of major discrepancy to which the test did not contribute (GIST to spindle cell RMS); and (7) 2 for* JAZF1-SUZ12* gene fusion, with 1 major discrepancy (pleomorphic sarcoma to endometrial stromal sarcoma (ESS)) and 1 minor discrepancy in which the test was not contributory (metastatic sarcoma to metastatic undifferentiated neoplasm). Overall, there were 7 cases in which FISH or PCR techniques contributed to the final diagnosis which resulted in either major or minor discrepancies.

## 4. Discussion

In our previous audit of referral cases to our institution in 2005, there were 349 specimens from 277 patients [4], of which diagnostic agreement was seen in 73.4% and diagnostic discrepancy in 27.5% (15.7% minor, 10.9% major, and 5% of the discrepant cases being benign-malignant discordances). In comparison, two decades, previously the Southeastern Cancer Study Group, reported a 28% disagreement rate between primary institutional diagnosis and reviewer diagnosis [7]. In 1989, the Scandinavian Sarcoma Group reported that 25% of reviewed sarcomas were reclassified, with grade changed in 40% [8]. The North West England peer review in 1991 showed a discrepancy rate of approximately 35% (disagreement in subtype in 17% and change in diagnosis to nonsarcomatous tumours in 18%), with an agreement rate of sarcoma subtype of 53%, and the remaining cases accounted for tumours where subtype could not be further specified, where classification was only possible as “malignant tumour NOS,” or where diagnosis could not be given [9]. The 2001 audit of soft tissue second opinion cases by Arbiser et al. showed major discrepancy in 25% of cases and minor discrepancy in 7% [10]. Finally, a three-centre analysis of French and Italian referrals showed a concordance rate of 56%, partial concordance of 35%, and complete discordance of 8% [11]. In our current study, there were similar frequencies of discrepancy to those described in our previous audit and to the other studies above. The slight variations encountered might be due to different criteria for classification of discrepancies. For example, we placed tumours which were not graded within the concordant group, as no error was actually made by the referring pathologist, but other studies have categorised these as minor discrepancies. We wished in particular to compare our current results with our previous findings, using identical criteria to define discrepancies. While the overall frequency of discrepant cases here (28.2%) was similar to our previous audit (26.6%) [4], in this study, we found more major discrepancies (16.4% in 2011 compared with 10.9% in 2005) and fewer minor discrepancies (11.8% in 2011 compared with 15.7% in 2005) compared with 2005. Furthermore, this increase in proportion of major discrepancies appeared to be mainly due to the number of benign-malignant discordances (23.5% compared with 5%).

This might in part be due to the increasing use of ancillary molecular and molecular cytogenetic testing, which has become commonplace in the diagnosis of soft tissue neoplasms since the previous study of 2005. A recent study assessing impact of molecular analysis on final sarcoma diagnosis in 763 cases found that such ancillary tests contributed to diagnosis of up to 4% of GISTs and 31% of ALT/WDL/DDL [12]. At our centre, the routine molecular and cytogenetics service for soft tissue sarcomas was established in 2006. In 2011, the year of this study, the service performed 405 FISH and 270 PCR analyses for soft tissue neoplasms. Of these, only 50 tests were performed on our referral cohort and contributed to 7 discrepancies. The most useful tests in reevaluating diagnosis were FISH (for assessing* MDM2* amplification status to determine whether differentiated lipomatous tumours or pleomorphic tumours were ALT/WDL or DDL, resp.) and for assessment of* EWSR1* gene rearrangement (for diagnosis of a variety of tumours, such as clear cell sarcoma).

However, this is still insufficient to explain the increase in proportion of major discrepancies, especially benign-malignant discordances. In our original audit, the commonest cause of discrepancies was found to be due to differences between the referring pathologist's interpretation of morphology or immunophenotype and that of the tertiary centre pathologist's, rather than the lack of or inappropriate use of immunohistochemical tests at the referring centre. Similarly, in this study, we found that the majority of referral cases had appropriate immunohistochemistry tests performed but that, in 23 cases, additional immunohistochemical tests contributed to the cases of diagnostic discrepancy of which the commonest antibodies omitted at referral centres were h-caldesmon for leiomyosarcoma, CDK4 for WDL/DDL, DOG1 for GIST, and beta-catenin for fibromatosis. It can certainly be argued that CDK4 is not sufficiently in widespread use in general pathology laboratories, although the other three antibodies, as well as others contributing to discrepancy, are in common use in the majority of diagnostic laboratories. It therefore appears that most discrepancies uncovered by immunohistochemistry are interpretational, due to unfamiliarity by the referring pathologist of either specific disease entities or antibodies, rather than due to the use by the tertiary centre of crucial rare antibodies not in common use in most laboratories.

Looking more specifically at discordant cases, there were fewer discrepancies related to GIST compared with 2005. Previously, pitfalls in the diagnosis of GIST existed due to inconsistent staining with CD34 and CD117 [13, 14], but now there is increasing familiarity with this neoplasm and its pattern of immunohistochemical staining, including the use of DOG1 antibody [15] as recommended in national guidelines. In contrast, there was no significant decrease in discrepancies involving leiomyosarcomas, fibromatosis, and liposarcomas, which were among the commonest causes of discrepancy. While in some cases this was due to lack of use of antibodies such as h-caldesmon and beta-catenin, in many the appropriate antibodies were used, but interpretation of morphologic features and immunohistochemical staining patterns led to discordant referring and tertiary centre diagnoses. Common sources of error included (a) diagnosing leiomyosarcoma based on focal expression of smooth muscle actin (SMA) and desmin alone, without the use of more specific smooth muscle markers [16] (as SMA can be diffusely expressed in myofibroblasts in both reactive and neoplastic conditions, and desmin is a broad spectrum marker of muscle lineage and is also expressed in other lesions such as myofibroblastoma), (b) interpreting cytoplasmic and especially paranuclear beta-catenin staining as positive for fibromatosis [17], and (c) interpreting overstaining or background staining of some antibodies, such as cytokeratins or CD31.

Therefore, in the majority of cases, as with our previous study, the major cause of discrepancies appears attributable to differences in interpretation by the referral and tertiary centre pathologists. The particular increase in benign-malignant interpretational differences might, in turn, be a result of (a) the increasing impetus to refer potential soft tissue cases to specialist centres with a subsequent deskilling of general pathologists in working up these cases and (b) increasing pressure on pathologists to reduce laboratory costs and turn-around times. In summary, we found that while overall rates of histological diagnostic discrepancy between referring and tertiary centre have remained stable 6 years following the previous study, there has been an increase in the proportion of major discrepancies. While this study is of a time period in which molecular and molecular cytogenetic ancillary diagnosis are commonplace, access to these investigations at the tertiary centre only contributed to a small fraction of diagnostic discrepancies, while, as before, interpretational differences contributed to the largest proportion of discrepancies, and this might be in part due to increasing lack of expertise in this specialist area, in the age of subspecialisation.

## Figures and Tables

**Table 1:** Patient and tumour characteristics.

Patient/tumour characteristics	Total
Male	111 (38.8%)
Female	175 (61.2%)
Median age	57.5 years (range 2–96 years)
Tumor size	7 cm (range 1–37 cm)
Tumor location	
Intra-abdominal	125
Uterus	39
Vagina/vulva	5
Pelvic/perineum	14
Retroperitoneum	13
Stomach	7
Small bowel	5
Colon/rectum	7
Mesenteries/peritoneum	13
Adrenal	1
Kidney	2
Bladder	5
Prostate	2
Liver	6
Abdomen NOS	6
Trunk	63
Breast	14
Chest wall	9
Abdominal wall/flank	9
Back	8
Buttock	6
Paraspinal region	6
Pubic	2
Sternum	1
Lower limb	45
Thigh	21
Groin/spermatic cord/scrotum	6
Knee	2
Calf/shin	5
Foot	6
Leg (not otherwise specified, NOS)	5
Upper limb	23
Axilla	4
Shoulder	8
Forearm	3
Wrist/finger	2
Arm NOS	6
Head and neck	23
Scalp	6
Orbit	2
Parotid	2
Nose	1
Cheek	3
Maxilla	1
Mandible	2
Oral/tongue	2
Neck	4
Thoracic cavity	11
Mediastinum	1
Trachea	1
Heart	1
Lung	8
Others (lymph nodes/skin/bone marrow)	4

NOS: not otherwise specified.

**Table 2:** Summary of cases showing minor discrepancy.

Referral diagnosis	Final diagnosis	*n*
Malignant	Malignant	

DFSP	DFSP with fibrosarcoma	5
DFSP with fibrosarcoma	Fibrosarcoma	1
DFSP with fibrosarcoma	Spindle cell sarcoma	1
Malignant SFT	Fibrosarcoma	1
Fibrosarcoma	MPNST	1
Myxoinflammatory fibrosarcoma	Myxofibrosarcoma	1
Myxoid liposarcoma	Myxofibrosarcoma	1
Spindle cell sarcoma	Myxofibrosarcoma	1
RMS NOS	Embryonal RMS	1
RMS NOS	Pleomorphic RMS	1
RMS NOS	Myoid sarcoma	1
Myoid sarcoma	Pleomorphic RMS	1
Leiomyosarcoma	Myoid sarcoma	4
Leiomyosarcoma	Spindle cell sarcoma	1
Leiomyosarcoma	Undifferentiated neoplasm	1
Leiomyosarcoma	Myofibrosarcoma	1
MPNST	Clear cell sarcoma	1
Undifferentiated neoplasm	Clear cell sarcoma	1
PEComa, ?atypical features	PEComa, malignant	2
DDL	Spindle cell sarcoma	1
Sarcoma	Malignant neoplasm	2
Extraskeletal myxoid chondrosarcoma	Malignant neoplasm	1

Benign	Benign	

Spindle cell lesion	Myxoma	1
Myxoid lesion	Myxoma	1
Benign neoplasm	Ossifying fibromyxoid tumour	1
Spindle cell lesion	Neurofibroma	1
Giant cell tumour of tendon sheath	Ossifying fibroma	1
SFT	Schwannoma	1
Fibrosis	Benign smooth muscle tumour	1

Grade	Grade	

GIST 2	GIST 3	1
GIST 3	GIST 2	1
Spindle cell sarcoma 3	Myxofibrosarcoma 2	1

DFSP: dermatofibrosarcoma protuberans; DDL: dedifferentiated liposarcoma; GIST: gastrointestinal stromal tumour; MPNST: malignant peripheral nerve sheath tumour; NOS: not otherwise specified; RMS: rhabdomyosarcoma; SFT: solitary fibrous tumour.

**Table 3:** Summary of cases showing major discrepancy.

Referral diagnosis	Final Diagnosis	*n*
Benign	Malignant (Including locally aggressive, although non-metastasising neoplasms)	

Angiomyxoma	Fibromatosis	1
Nerve sheath tumour	Fibromatosis	1
Granular cell tumour (benign)	Granular cell tumour (malignant)	4 (1 patient)
Haemangiopericytoma	DDL	1
Leiomyoma	Leiomyosarcoma	2
Reactive tissue	Leiomyosarcoma	1

Malignant (Including locally aggressive although non-metastasising neoplasms)	Benign	

Fibromatosis	Scar	1
Fibromatosis	Nuchal-type fibroma	1
ALT	Lipoma	2
MPNST	Atypical neurofibroma	1
Leiomyosarcoma	BFH	1
DFSP	BFH	1
Metastatic renal carcinoma	Clear cell BFH	1
Angiosarcoma	Haemangioma	1
GIST	Fibrous tissue only	1
Leiomyosarcoma	Schwannoma	1
IMT	Wegener's granulomatosis	1

Malignant	Malignant	

STUMP	Leiomyosarcoma (grade 2)	2
ESS	Leiomyosarcoma	1
ESS	Osteosarcoma	1
GIST	Spindle cell sarcoma	1
GIST	Spindle RMS	1
Leiomyosarcoma	MPNST	1
LGFMS	Fibromatosis	1
LGFMS	DDL	1
Synovial sarcoma	Malignant neoplasm	1

Benign	Benign	

Schwannoma	Benign naevus	1

Mesenchymal	Non-mesenchymal	

UPS	Carcinosarcoma	4
Synovial sarcoma	Carcinosarcoma	1
Chondrosarcoma	Carcinosarcoma	1
Leiomyosarcoma	Carcinosarcoma	1
Sarcoma	Seminoma	1
MPNST	Melanoma	2
Non-mesenchymal	Mesenchymal	

Carcinoma	Pleomorphic RMS	1
Carcinoma	Spindle cell sarcoma	1
Lymphoma	SFT	1

Non-mesenchymal	Non-mesenchymal	

Undifferentiated carcinoma	Prostatic carcinoma	1

Grade	Grade	

GIST grade 2/3	GIST 1	2
Leiomyosarcoma 2/3	Leiomyosarcoma 1	2
Leiomyosarcoma 1	Leiomyosarcoma 2	1
Myxofibrosarcoma 2	Myxofibrosarcoma 1	1
WDL	DDL grade 2	1
High grade sarcoma	ESS	1

Abbreviations: ALT atypical lipomatous tumour; BFH benign fibrous histiocytoma; DFSP dermatofibrosarcoma protuberans; DDL dedifferentiated liposarcoma; ESS endometrial stromal sarcoma; GIST gastrointestinal stromal tumour; IMT inflammatory myofibroblastic tumour; LGFMS low grade fibromyxoid sarcoma; MPNST malignant peripheral nerve sheath tumour; RMS rhabdomyosarcoma; SFT solitary fibrous tumour; STUMP smooth muscle tumour of uncertain malignant potential; UPS undifferentiated pleomorphic sarcoma; WDL well differentiated liposarcoma.
